# AMPK-ULK1-Mediated Ferritinophagy Drives Ferroptosis in GLA-Induced Testicular Toxicity

**DOI:** 10.34133/research.0860

**Published:** 2025-06-25

**Authors:** Dianyun Wang, Caiying Zhang, Fan Yang, Yang Hu, Chenghong Xing, Guoliang Hu, Jirong Chen, Yi Li, Penghui Liu, Huabin Cao, Xueyan Dai

**Affiliations:** ^1^ Jiangxi Provincial Key Laboratory for Animal Health, Institute of Animal Population Health, College of Animal Science and Technology, Jiangxi Agricultural University, Nanchang, Jiangxi, China.; ^2^ College of Computer and Information Engineering, Jiangxi Agricultural University, Nanchang, Jiangxi, China.

## Abstract

The health problem of infertility has garnered increasing attention, prompting a deeper understanding of its causes. The broad-spectrum and nonselective herbicide glufosinate ammonium (GLA) is widely used in many countries. Previous studies have demonstrated the reproductive toxicity of GLA, but its potential toxic mechanisms remain unclear. Here, mice, Sertoli cells, and Leydig cells were used to create GLA preconditioning models. Results showed that GLA exposure caused morphological and functional damage of sperm. Concurrently, our study revealed that GLA, similar to Erastin, could induce ferroptosis in Sertoli and Leydig cells, as indicated by the dose-dependent increases of intracellular iron levels, lipid peroxidation, and cell death. Additionally, both the lipid ROS scavenger Fer and the iron chelator deferiprone were found to mitigate GLA-induced cell death. Intriguingly, our findings suggested that GLA-induced ferroptosis was dependent on autophagy, as the use of pharmacological inhibitors (3-methyladenine, chloroquine, and bafilomycin A1) or autophagy-related gene 5 gene knockout markedly reduced ferroptosis induced by GLA. We also demonstrated that nuclear receptor coactivator 4 (NCOA4)-mediated ferritinophagy, which involves the autophagic degradation of the primary intracellular iron storage protein ferritin, is essential for GLA-induced ferroptosis by showing that NCOA4 knockdown decreased intracellular iron levels and attenuated lipid peroxidation, eventually alleviating GLA-induced cell death. Moreover, we observed that inhibition of the AMP-activated protein kinase–Unc-51-like kinase 1 (AMPK-ULK1) pathway activity by knockdown of AMPK expression markedly reduced the mitochondrial reactive oxygen species (mtROS) level and alleviated GLA-induced ferroptosis. Collectively, GLA induced excessive mtROS production through activation of the AMPK-ULK1 pathway, triggering excessive autophagy that ultimately led to ferroptosis via NCOA4-mediated ferritinophagy.

## Introduction

Growing evidence indicates that agricultural pesticide contamination poses a global challenge to environmental ecosystems and human health [1–3]. Pesticides, which are utilized for the management of pests and weeds, can be classified into a range of categories including herbicides, insecticides, rodenticides, and bactericides. Among these, herbicides account for approximately 40% of total pesticide usage [4]. Consequently, glufosinate ammonium (GLA) is being extensively used, and its utilization is increasing worldwide due to its characteristics of phytotoxicity, which takes effect rapidly [5]. However, given the fact that GLA is highly soluble in water (>500 g/l), it can easily be transferred to the aquatic environment [6]. Relevant data show that GLA concentrations in the Musoncello and Teva Rivers exceed the European upper limit for pesticides in river water (0.1 μg/l) [7]. Similarly, GLA concentrations in the waters of 10 provinces in China range from 2.380 to 13.150 μg/l, notably surpassing the limit of 0.100 μg/l [8]. This situation poses a considerable risk to aquatic life and may threaten the fundamental health of animals and humans through bioaccumulation in the food chain. Mounting evidence suggests that GLA is detected in human specimens and caused multiple organ systems injury, with the reproductive organs being one of the target organs [9–11]. However, the comprehensive toxicological characteristics and mechanisms of GLA-induced cytotoxicity remain to be fully understood.

In recent decades, male fertility has been declining due to dietary behaviors, lifestyle factors, and environmental chemicals [12]. Studies have shown that these pesticides can damage the testis and contribute to decreased male fertility including organophosphates, pyrethroids, methylenedioxyphenyl, and organochlorines [13]. Leydig and Sertoli cells are major components of the testis, each with distinct functions. Sertoli cells supply vital nutrients to germ cells and play a crucial role in spermatogenesis. Leydig cells, located in the interstitial regions surrounding the seminiferous tubules, serve as the primary source of androgens [14]. Injury to either Leydig or Sertoli cells can result in remarkable pathological changes within the reproductive system [15]. According to da Costa et al. [16], chlorpyrifos exposure during the peripubertal period in rats can impair sperm parameters and reduce the quantity of both Sertoli and Leydig cells. Additionally, Xiao et al. investigated how adolescents were impacted by exposure to environmentally relevant levels of the organophosphate insecticide malathion. Their findings indicated that malathion exposure can lead to spermatogenic dysfunction by stimulating the hypoxia-inducible factor 1/mitogen-activated protein kinase/phosphatidylinositol 3-kinase (HIF-1/MAPK/PI3K) pathway [17]. It is conceivable that GLA can infiltrate Leydig and Sertoli cells through ingestion, occupational exposure, or accidental inhalation, potentially leading to or exacerbating testicular diseases. Nonetheless, there is currently no evidence regarding whether GLA exhibits cell type-specific effects on the testis. Meanwhile, the underlying mechanisms responsible for GLA-induced testicular toxicity remain unclear.

Ferroptosis, a regulated cell death mechanism, is marked by conspicuous lipid peroxidation, primarily due to high iron levels and the generation of reactive oxygen species (ROS). Glutathione peroxidase 4 (GPX4) is essential for converting harmful lipid hydroperoxides into safe lipid forms. Inactivation of GPX4 results in the buildup of lipid hydroperoxides, leading to ferroptosis induction [18]. The SLC7A11 gene, which codes for the xCT system transporter, is fundamental in the “iron overload-ferroptosis” pathway [19]. Acyl-CoA synthetase long-chain family member 4 (ACSL4) increases polyunsaturated fatty acid (PUFA) levels in phospholipids, making them more prone to ferroptosis [20]. These proteins are recognized as biomarkers of ferroptosis. Ferroptosis is closely governed by iron metabolism. Ferritin, the principal iron storage protein, consists of 2 subunits: light chain (FTL) and heavy chain 1 (FTH1) [21]. Activating autophagy can lead to ferritin degradation, increasing intracellular iron levels and causing oxidative damage via the Fenton reaction. Nuclear receptor coactivator 4 (NCOA4) mediates ferritin’s autophagic clearance in lysosomes, a process termed ferritinophagy [22]. Studies have emphasized the vital function of ferroptosis in the development of a wide range of disorders, including premature ovarian insufficiency, primary ovarian insufficiency, and oligospermia [23–26]. Recent research has also recognized ferroptosis as a potential mechanism underlying the toxicity induced by various agents. Specifically, the association between reproductive toxicity from pesticide exposure and ferroptosis has been highlighted. According to the research, di(2-ethylhexyl) phthalate (DEHP) triggers ferroptosis, leading to blood–testis barrier dysfunction through the interaction with the transferrin receptor in mouse testicular tissues [27]. Additionally, Cheng et al. [28] identified heme oxygenase-1 (HO-1), which mediates the ferroptosis triggered by diquat dibromide in mouse spermatogonial cells. Therefore, we hypothesized that ferroptosis contributes to the testicular toxicity induced by GLA in mouse Sertoli and Leydig cells.

In the current research, we identified the toxic impacts of GLA in the mice testis, Sertoli cells, and Leydig cells. Our in vivo findings suggested that GLA exposure led to testicular tissue damage and ferritinophagy in mice. Furthermore, we demonstrated that GLA-triggered autophagy was essential for NCOA4-mediated ferritin degradation, which is closely linked to the ferroptosis induced by GLA in both Sertoli and Leydig cells. Simultaneously, we uncovered that activation of the AMPK-ULK1 signaling pathway contributes to the regulation of GLA-induced ferroptosis in these cell types. However, supplementation with ferroptosis inhibitors and knockdown of NCOA4 remarkably reversed GLA-induced Sertoli cells and Leydig cells injury. Our findings initially highlighted that the ferroptosis of Sertoli and Leydig cells was a key molecular mechanism underlying GLA-induced testicular toxicity, and provide essential reference values for the prevention and treatment of testicular toxicity induced by GLA exposure.

## Results

### GLA exposure causes mice testicular injury

**Fig. 1.**
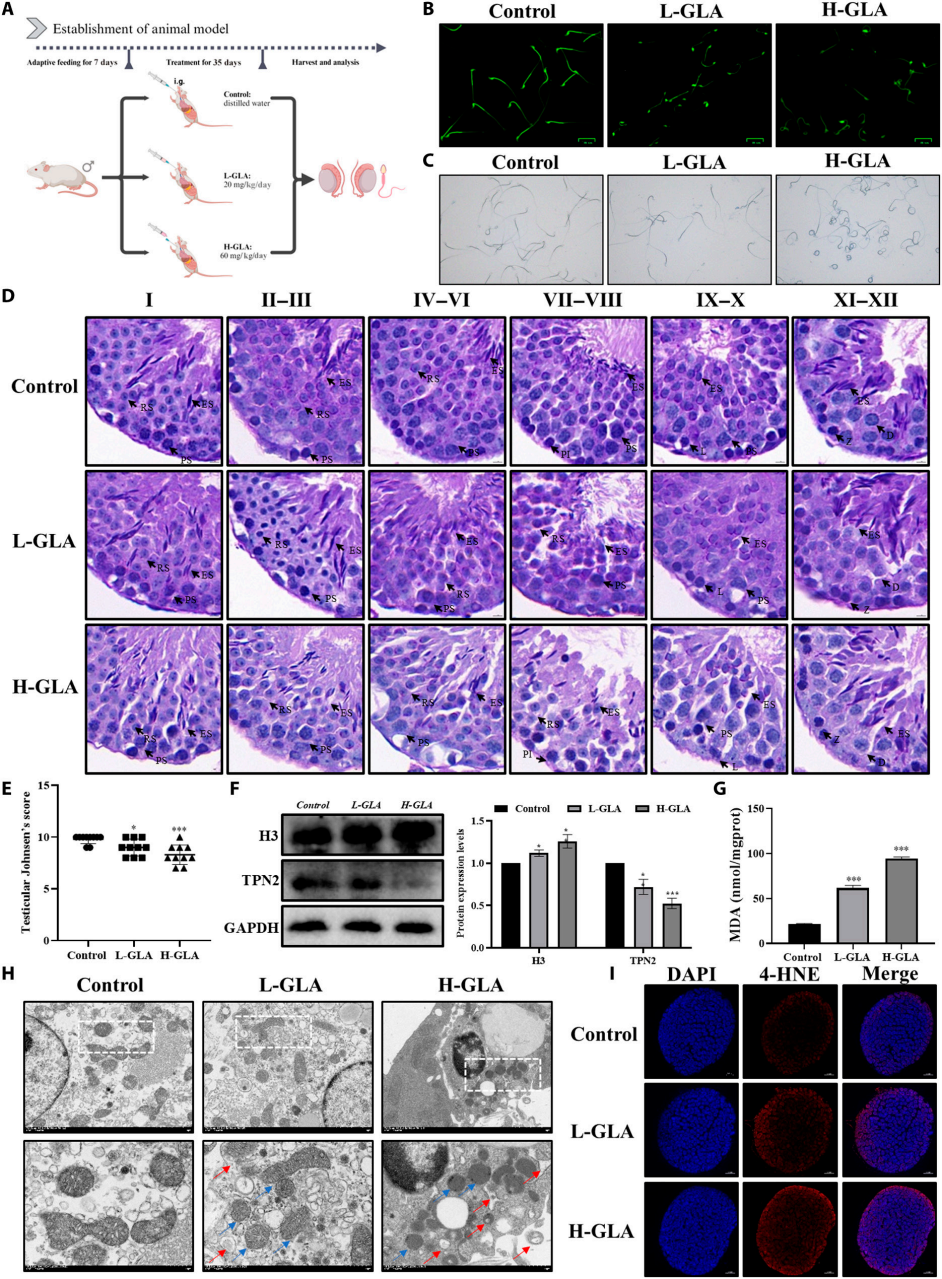
GLA exposure causes mice testicular injury. Schematic of the experimental design for the intragastric administration to establish a GLA exposure model (A). Acridine orange staining in testes following 20 or 60 mg/kg GLA treatment (B). Aniline blue staining (C). PAS staining (D). Testicular Johnsen’s score (E). The protein expression levels of spermatogenic factors (F). MDA content (G). Observation of the ultrastructure: blue arrows indicate mitochondria with severely disrupted cristae, and red arrows indicate mitochondria vacuolation (H). The immunofluorescent staining of 4-HNE (I). Data are presented as mean ± SD of at least 3 independent experiments. “*” indicates significant difference compared with the control group (*P < 0.05 and ***P < 0.001).

To examine the impact of GLA on male reproductive function, we developed a mice model exposed to GLA (Fig. 1A). Male fertility is contingent upon the production of numerous high-quality sperm during spermatogenesis. To evaluate sperm quality following GLA exposure, we stained sperm with acridine orange to assess DNA damage. The results showed that sperm in the control group exhibited clear and uniform green fluorescence. In contrast, after exposure to increasing doses of GLA, the fluorescence became less uniform and diminished, suggesting DNA damage in the sperm following GLA treatment (Fig. 1B). Additionally, aniline blue staining revealed lighter staining of sperm in the control group, which deepened with increasing GLA concentrations, suggesting impaired sperm maturation due to GLA treatment (Fig. 1C). Importantly, GLA treatment also induced morphological changes in sperm. Subsequently, we employed periodic acid–Schiff (PAS) staining to investigate the impact of GLA on testicular function, particularly spermatogenesis. Compared with the control group, seminiferous tubules display structural disruption, looseness, disarray, and indistinctness, with disordered germinal epithelium morphology, thinning and disruption of the germ cell layer, and decreased sperm count in the lumen, accompanied by a marked decline in Johnsen’s score in the GLA group (Fig. 1D and E). Furthermore, the protein expression levels associated with spermatogenic function, particularly those of TNP2, were substantially decreased in the GLA group. Conversely, the protein level of H3 was distinctly elevated (Fig. 1F). These findings jointly suggested that GLA exposure exerted damage on sperm quality in a dose-dependent manner.

Ultrastructural analysis revealed extensive mitochondrial vacuolization and cristae loss in Sertoli cells, along with increased autophagic vacuoles (Fig. 1G). Considering that GLA inhibits glutamine synthetase (GS), it leads to elevated levels of ROS, an increase in the lipid peroxidation level, and damage to cell membranes; malondialdehyde (MDA) and 4-hydroxynonenal (4-HNE) were selected as key indicators [29]. The MDA content showed a pronounced increase in the GLA-treated groups (Fig. 1H); meantime, the fluorescence intensity of 4-HNE increased substantially (Fig. 1I). Collectively, these findings highlighted that GLA causes testicular damage in mice by impairing spermatogenesis and maturation, inducing mitochondrial injury, and elevating lipid peroxidation levels in a dose-dependent manner.

### GLA exposure induces damage to Sertoli cells and Leydig cells

Sertoli cells and Leydig cells in mouse testes play crucial roles in maintaining normal reproductive function. Therefore, we used Sertoli and Leydig cells to study the toxic effects of GLA exposure on testes. Initially, we assessed the IC_50_ values in the cells. Exposure to different GLA concentrations for a 24-h period indicated that the IC_50_ concentration for Sertoli cells is 1,662 μmol/l, while for Leydig cells, it is 2,027.5 μmol/l (Fig. S1A). Subsequently, cells were treated with GLA concentrations of 1/10, 1/8, 1/6, and 1/4 of the IC_50_ for 24 h to assess cell viability. Sertoli cells showed no marked change in viability at the GLA concentrations of 1/10, but it exhibited a gradual decline at the GLA concentrations of 1/8. Conversely, Leydig cells showed no notable change in viability at the GLA concentrations of 1/8, but displayed a gradual decline at the GLA concentrations of 1/6 (Fig. S1B). Therefore, we selected GLA concentrations of 1/10, 1/8, and 1/6 of the Sertoli cell IC_50_ as exposure concentrations, and 1/8, 1/6, and 1/4 of the Leydig cell IC_50_ as exposure concentrations, respectively. After this, we applied the highest concentration to cells for 0, 6, 12, and 24 h, observing a pronounced decline in cell viability at 24 h (Fig. S1C).

An ultrastructural analysis after 24 h of GLA treatment revealed that both Sertoli and Leydig cells exhibited mitochondrial swelling, membrane rupture, cristae disruption, and the assembly of autophagic vesicles (Fig. S2A). Additionally, the MDA contents increased with increasing GLA concentrations in both cells (Fig. S2B). Mitochondrial membrane potential (MMP) and mitochondrial reactive oxygen species (mtROS) are commonly used to evaluate mitochondrial function. The results showed that GLA treatment resulted in a reduction in MMP and an elevation in mtROS (Fig. S2C and D). These findings collectively suggested that GLA exposure leads to damage to Sertoli and Leydig cells. In addition, YO-PRO-1/PI staining demonstrated that GLA treatments increase the proportion of PI-positive/YO-PRO-1-positive cells (Fig. S2E), suggesting that GLA may induce a form of cell death, which warrants further investigation.

### GLA exposure induces ferroptosis

**Fig. 2.**
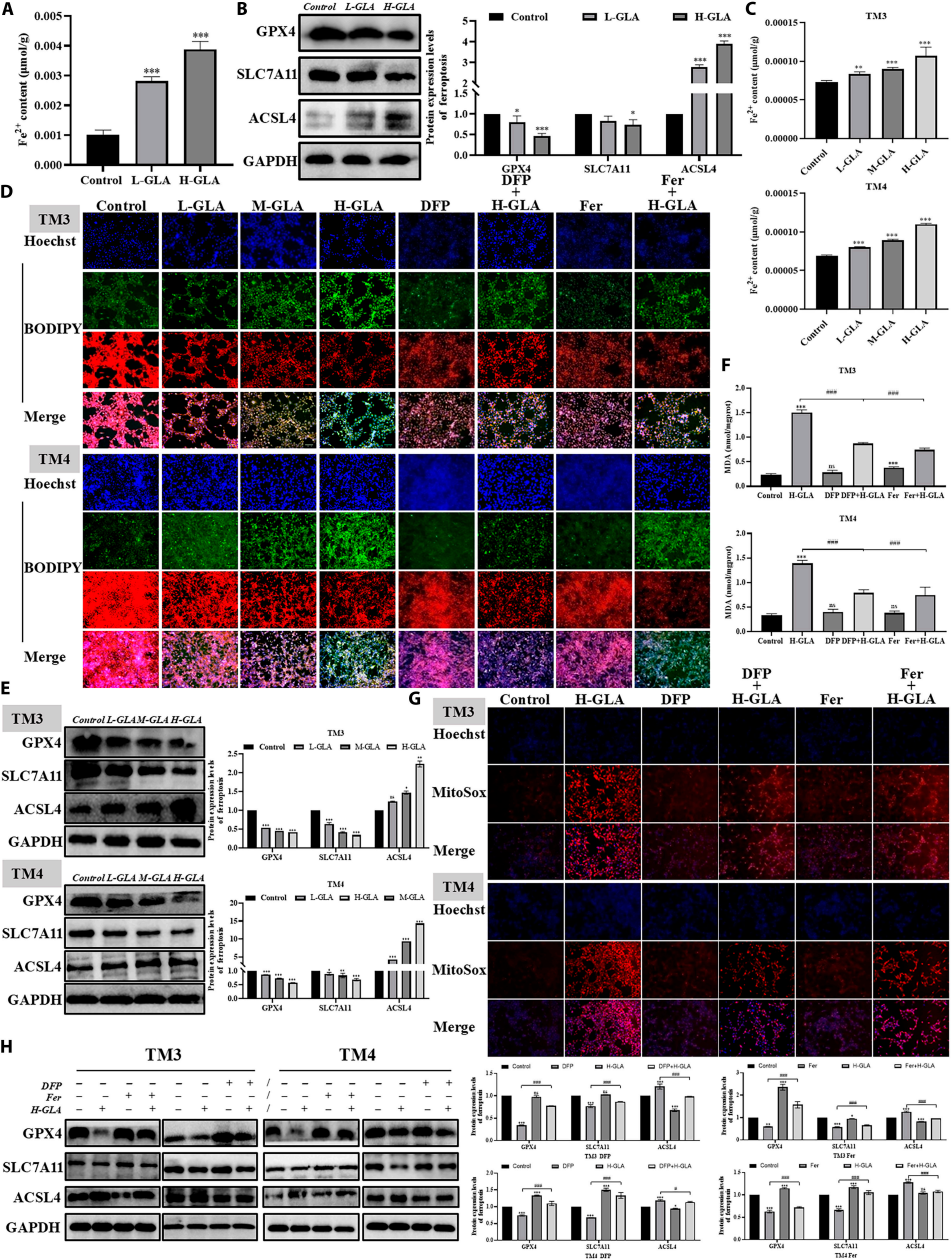
GLA exposure induces ferroptosis in mouse testes. The Fe^2+^ content of testes tissues (A). The protein expression levels of GPX4, SLC7All, and ACSL4 in testes tissues (B). The Fe^2+^ content of Sertoli cells and Leydig cells (C). Representative fluorescence images of lipid peroxidation assay in Sertoli cells and Leydig cells following GLA treatment for 24 h combined with Fer-1 (200 nM) or DFP (6 or 4 μM) (D). The protein expression levels of GPX4, SLC7All, and ACSL4 of Sertoli cells and Leydig cells (E). MDA content (F). Representative fluorescence confocal images of mROS with MitoSox dye in Sertoli cells and Leydig cells following GLA treatment for 24 h combined with Fer-1 or DFP (G). The protein expression levels of GPX4, SLC7All, and ACSL4 in Sertoli cells and Leydig cells following GLA treatment for 24 h combined with Fer-1 or DFP (H). Data are presented as mean ± SD of at least 3 independent experiments. “*” indicates significant difference compared with the control group (*P < 0.05, **P < 0.01, and ***P < 0.001). “^#^” indicates significant difference between the GLA treatment group and combined treatment group (^#^*P* < 0.05 and ^###^*P* < 0.001).

Given that the key features of ferroptosis involve iron accumulation, lipid peroxidation, and changes in mitochondrial morphology, our previous experimental data have demonstrated that GLA causes mitochondrial damage and increased lipid peroxidation. To further investigate the mechanism by which GLA causes testicular damage in mice, we initially measured the levels of ferrous ions in testicular tissues. The findings indicated a substantial increase in ferrous ion content in the GLA-treated group, which was positively correlated with the supplementation dose compared with the control group (Fig. 2A). Simultaneously, the data showed that the levels of GPX4 and SLC7A11 notably declined and the levels of ACSL4 substantially rose in a dose-dependent manner in the GLA-treated group (Fig. 2B). Furthermore, immunohistochemical results indicated that GLA treatment increased GPX4 expression in testicular tissue (Fig. S3A). Additionally, detection of GSH content in the tissue revealed that GLA treatment elevated testicular GSH levels (Fig. S3B). These data communally suggested that GLA exposure triggers ferroptosis in mouse testicular tissues.

Previous research has demonstrated that specific cell types may exhibit sensitivity or resistance to ferroptosis [30]. In this study, we aimed to investigate whether Sertoli cells and Leydig cells exhibit sensitivity or resistance to ferroptosis. Our experiments illustrate that compared with normal cells, GLA treatment notablely increased intracellular ferrous ion content (Fig. 2C) and the oxidized state fluorescence level of lipid peroxidation, decreased the reduced state fluorescence intensity (Fig. 2D) and the level of GPX4 and SLC7A11, but increased the ACSL4 protein level (Fig. 2E). As a preeminent ferroptosis inducer, Erastin has been extensively employed in fundamental research on ferroptosis mechanisms. To further validate the ferroptotic effect of GLA, we utilized Erastin as a positive control to induce ferroptosis in Sertoli and Leydig cells (Fig. S3C). Following 24 h treatment with Erastin (200 or 100 nM) and H-GLA, both the Fe^2+^ concentration (Fig. S3D) and lipid peroxidation levels (Fig. S3E) in the Erastin positive control group and H-GLA group increased markedly compared with the control group. Additionally, Western blot (WB) results indicated that Erastin and H-GLA treatment led to reduced expression of GPX4, SLC7A11, and FTH1 proteins, along with increased levels of ACSL4 and NCOA4 proteins (Fig. S3F). These data indicated that Sertoli and Leydig cells are sensitive to ferroptosis.

Following this, we investigated whether the inhibition of ferroptosis could mitigate cell death induced by GLA exposure. Ferrostatin-1 (Fer-1) was acknowledged as the most effective agent in preventing ferroptosis [31]; it specifically blocks lipid peroxidation without influencing the formation of mtROS and the stability of lysosomal membranes [32]. Additionally, deferiprone (DFP), a compound belonging to the hydroxypyrid-4-one class, was employed as an iron-binding agent in biological studies and has been explored for its therapeutic potential in treatment systemic iron overload diseases [33]. To better clarify the molecular mechanisms underlying GLA-induced ferroptosis, the cells were individually exposed to Fer-1 and DFP (Fig. S4A). Our findings indicated that both Fer-1 and DFP effectively suppress the accumulation of MDA in Sertoli and Leydig cells after exposed to GLA (Fig. 2F). Immunofluorescence data demonstrated that the addition of Fer-1 and DFP reversed the decrease in MMP (Fig. S4B) and the increase in lipid peroxidation (Fig. 2D) and mitochondrial ROS levels (Fig. 2G) induced by GLA. WB results indicated that Fer-1 and DFP treatment reversed the reduced expression of GPX4 and SLC7A11 proteins after GLA exposure, as well as elevated the ACSL4 protein level (Fig. 2H). These data suggested that GLA treatment triggered ferroptosis in Sertoli cells and Leydig cells, and inhibition of ferroptosis could mitigate GLA-induced cellular death.

### Autophagy was activated by GLA and was required for GLA-induced ferroptosis of Sertoli cells and Leydig cells

**Fig. 3.**
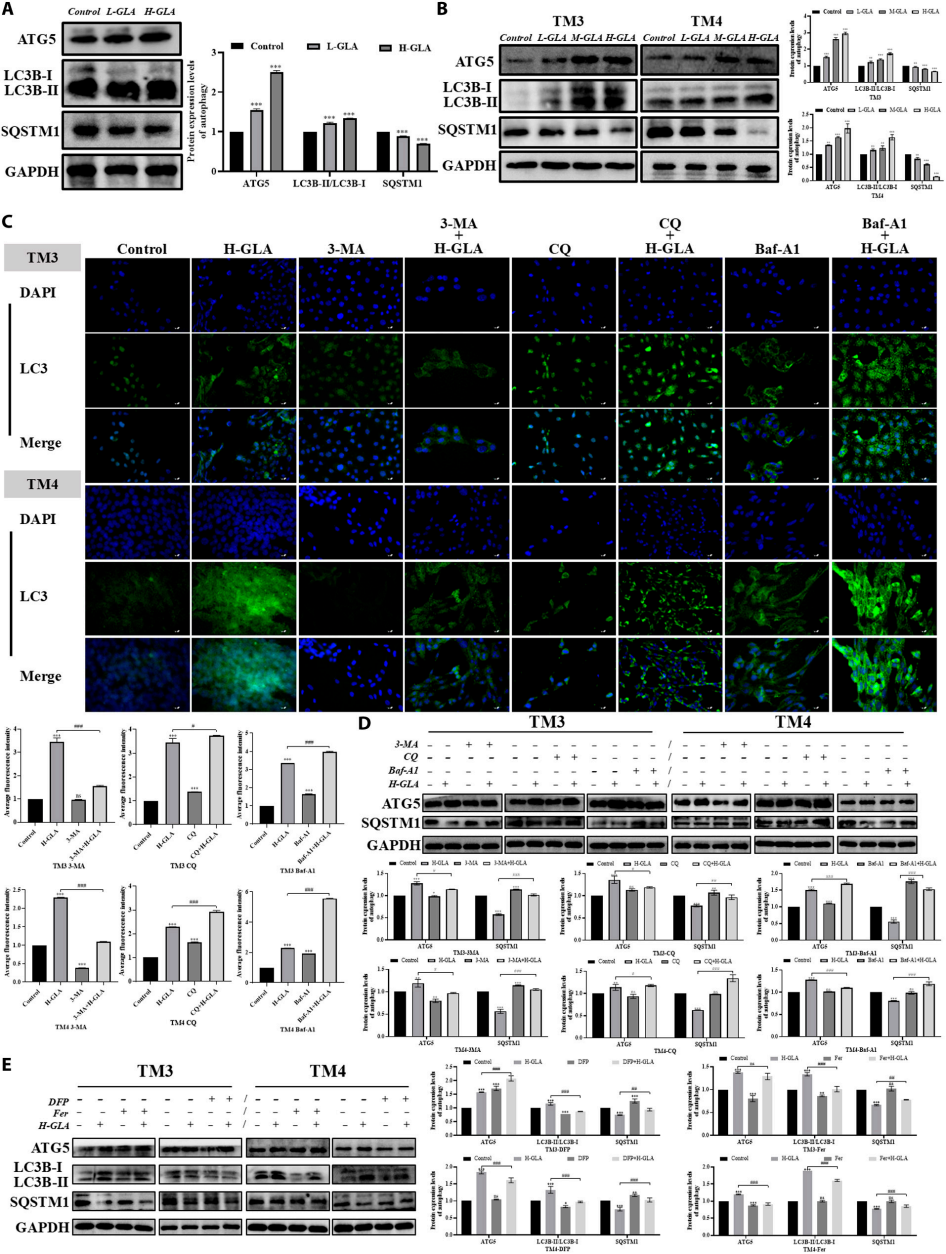
GLA exposure triggers mitophagy in mouse testes. The protein expression levels of ATG5, LC3B, and SQSTM1 in testes tissues (A) and Sertoli cells and Leydig cells (B). Representative fluorescence confocal images of endogenous LC3B in Sertoli cells and Leydig cells following GLA treatment for 24 h combined with 3-MA (60 or 90 μM), CQ (0.5 mΜ or 4 μM), or Baf-A1 (12 or 4 nM) and statistical analysis (C). The protein expression levels of ATG5, LC3B, and SQSTM1 in Sertoli cells and Leydig cells following GLA treatment for 24 h combined with 3-MA, CQ, or Baf-A1 (D). The protein expression levels of ATG5, LC3B, and SQSTM1 in Sertoli cells and Leydig cells following GLA treatment for 24 h combined with Fer-1or DFP (E). “*” indicates significant difference compared with the control group (*P < 0.05, **P < 0.01, and ***P < 0.001). “^#^” indicates significant difference between the GLA treatment group and combined treatment group (^#^*P* < 0.05, ^##^*P* < 0.01, and ^###^*P* < 0.001).

Ferroptosis is commonly associated with autophagy and considered a form of autophagy-dependent cell death [34]. Our ultrastructural results showed the presence of autophagosomes in testicular tissues after GLA treatment. Therefore, we examined the underlying pathways that contribute to ferroptosis triggered by GLA. In both in vivo and vitro experiments, GLA markedly enhanced the ratio of microtubule-associated protein 1 light chain 3B-II to LC3B-I (LC3B-II/LC3B-I) and the expression of autophagy-related gene 5 (ATG5), but distinctly down-regulated the protein level of sequestosome-1 (SQSTM1) (Fig. 3A and B). Meantime, autophagosomes were observed within the cells under transmission electron microscopy (TEM) images. These results indicated that GLA may activate autophagy in mice testicular in a dose-dependent manner.

To further verify these findings, we treated cells with autophagy inhibitors 3-MA, CQ, and Baf-A1 to investigate the effects of GLA on autophagy (Fig. S5A). We first co-treated cells with H-GLA and 3-MA, CQ, or Baf-A1 for 24 h, following by immunofluorescence detection of LC3 expression. The experiments illustrate that co-treatment with 3-MA and H-GLA reduced the LC3 expression, indicating that 3-MA inhibits the formation of GLA-induced autophagosomes. Conversely, H-GLA co-treatment respectively with CQ and Baf-A1 increased the LC3 expression (Fig. 3C). WB analysis of autophagy-related proteins results indicated that 3-MA alleviated the H-GLA-induced increase in ATG5 protein expression and the decrease in SQSTM1 protein expression, while co-treatment of H-GLA with CQ and Baf-A1 led to the accumulation of ATG5 and SQSTM1 (Fig. 3D). These findings indicated that GLA activated autophagy, and the inhibitors prevented the fusion and degradation of autophagosomes with lysosomes, leading to the accumulation of autophagosomes within the cells. Additionally, WB results also showed that H-GLA co-treatment respectively with Fer-1 and DFP treatment reversed the expression of autophagy-related proteins (Fig. 3E). These data suggested that GLA can trigger autophagy in Sertoli and Leydig cells in a dose-dependent manner and has a potential association with ferroptosis.

### NCOA4-mediated ferritin degradation is essential for ferroptosis in Sertoli and Leydig cells under GLA exposure

**Fig. 4.**
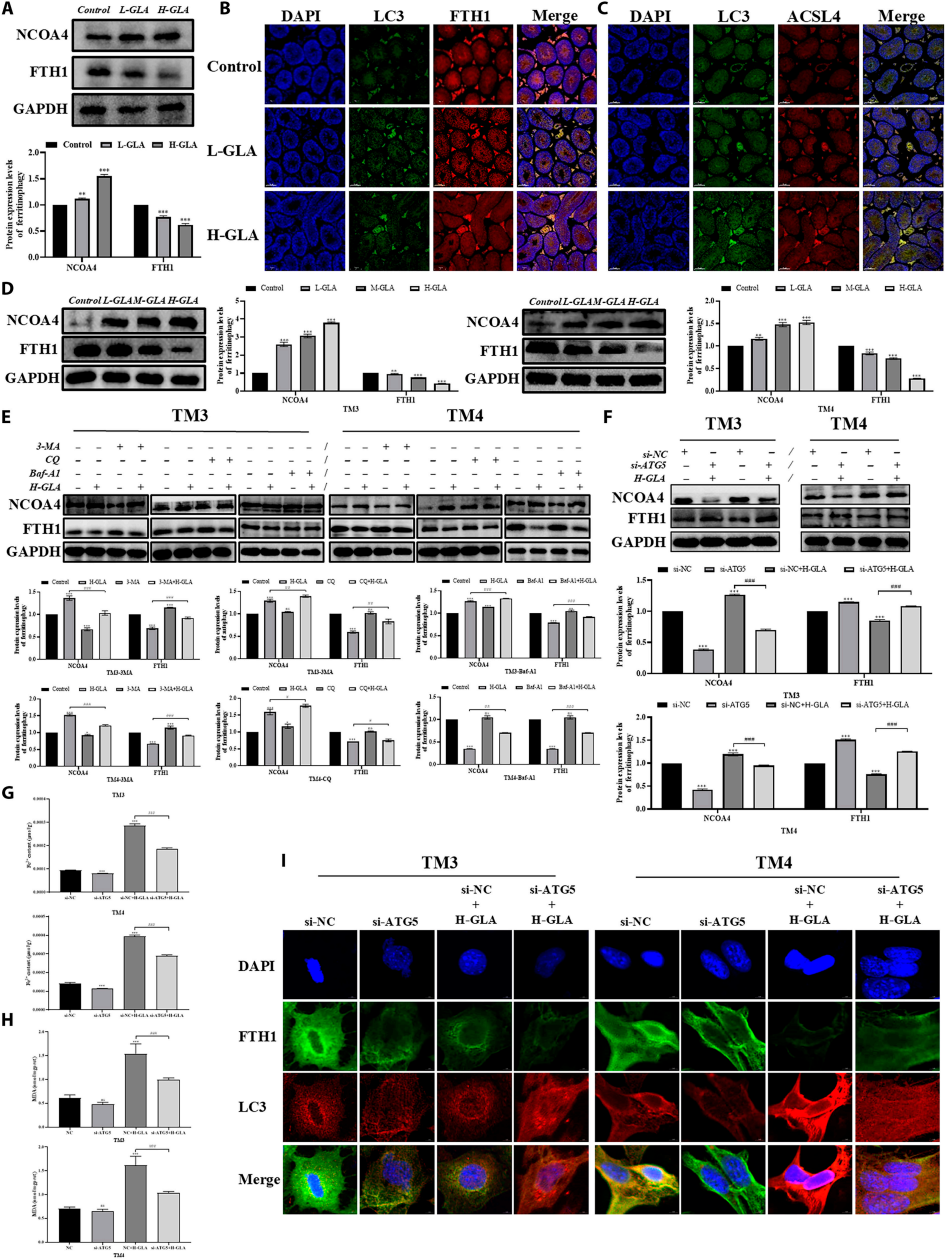
Autophagy was required for GLA-induced Sertoli cell and Leydig cell ferroptosis. The protein expression levels of NCOA4 and FTH1 in testes tissues (A). Representative fluorescence confocal images of LC3B and FTH1 colocalization in testes (B). Representative fluorescence confocal images of LC3B and ACSL4 colocalization in testes (C). The protein expression levels of NCOA4 and FTH1 in Leydig cells and Sertoli cells (D). The protein expression levels of NCOA4 and FTH1 in Sertoli cells and Leydig cells following GLA treatment for 24 h combined with 3-MA, CQ, or Baf-A1 (E). The protein expression levels of NCOA4 and FTH1 in Sertoli cells and Leydig cells following GLA treatment for 24 h combined with si-ATG5 (F). Fe^2+^ content (G). MDA content (H). Representative fluorescence confocal images of LC3B and FTH1 colocalization in Sertoli cells and Leydig cells following GLA treatment for 24 h combined with si-ATG5 (I). “*” indicates significant difference compared with the control group (*P < 0.05, **P < 0.01, and ***P < 0.001). “^#^” indicates significant difference between the GLA treatment group and combined treatment group (^#^*P* < 0.05, ^##^*P* < 0.01, and ^###^*P* < 0.001).

Autophagy activation can increase intracellular iron levels by degrading ferritin, which promotes oxidative damage via the Fenton reaction, a process known as ferritinophagy mediated by NCOA4. Ferritinophagy can specifically recognize and bind to the ferritin component heavy chain 1 (FTH1), and transports FTH1 to autophagosomes for lysosomal degradation, eventually causing the release of ferrous ions [35]. Excessive ferritinophagy can cause ferroptosis due to intracellular ferrous ion overload. Consequently, we quantified the NCOA4 and FTH1 protein expression levels. Relative to the control group, the NCOA4 protein level in the GLA-treated group increased observably, while the FTH1 protein level decreased markedly (Fig. 4A). Furthermore, our immunofluorescence colocalization results demonstrated that GLA reduced the colocalization of LC3 with FTH1 (Fig. 4B), but enhanced the colocalization of LC3 with ACSL4 (Fig. 4C). These observations confirmed that GLA activated ferritinophagy in mice testicular tissue. Subsequently, cell cultures were exposed to different GLA dosages over a period of 24 h, and the results showed that GLA prominently increased the NCOA4 protein expression and decreased the FTH1 protein expression (Fig. 4D). To further investigate, we co-treated cells with H-GLA and autophagy inhibitors 3-MA, CQ, and Baf-A1 for 24 h. WB results showed that 3-MA restored the expression levels of NCOA4 and FTH1, while CQ and Baf-A1 increased the protein expression levels of NCOA4 and FTH1 (Fig. 4E). Additionally, immunofluorescence colocalization results show that the addition of 3-MA decreased the colocalization of LC3 and FTH1, whereas the addition of CQ and Baf-A1 has an opposite result (Fig. S5B). These observations suggested that 3-MA inhibited autophagosome formation and thereby suppressed GLA-induced ferritinophagy. In contrast, CQ and Baf-A1 as autophagic lysosome inhibitor did not fully suppress GLA-induced ferritinophagy, which needs further investigation. Subsequently, we performed small interfering RNA (siRNA)-mediated knockdown of ATG5 using a selected siRNA sequence with 65% knockdown efficiency (Fig. S6A). Our findings revealed that knockdown of ATG5 effectively down-regulated the protein level of NCOA4, elevated the FTH1 protein expression (Fig. 4F), distinctly declined the Fe^2+^ content (Fig. 4G), and increased the MDA level (Fig. 4H). Additionally, knockdown of ATG5 markedly restored the GLA-induced decrease in ferritin levels and increased the colocalization of LC3 and FTH1 in GLA-treated cells (Fig. 4I). These results indicated that GLA-induced ferritinophagy in Sertoli and Leydig cells is attributed to the stimulation of autophagosome formation rather than autolysosome maturation. Additionally, inhibition of autophagosome formation mitigated ferritin deposition.

**Fig. 5.**
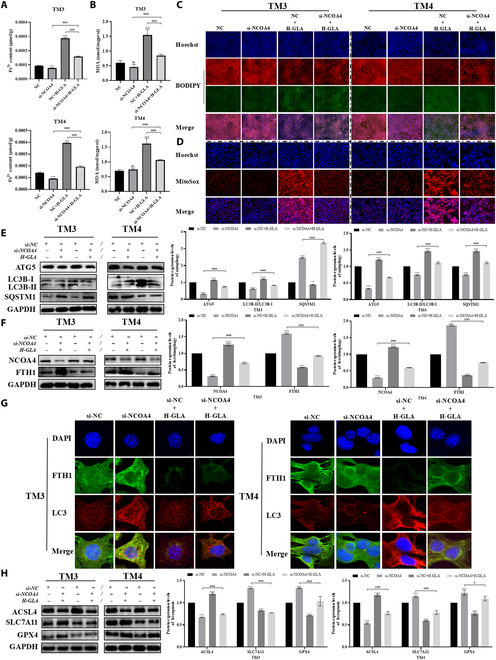
NCOA4-mediated ferritin degradation is essential for ferroptosis in Sertoli and Leydig cells under GLA exposure. The content of Fe^2+^ in Sertoli cells and Leydig cells following GLA treatment for 24 h combined with si-NCOA4 (A). MDA content (B). Representative fluorescence confocal images of lipid peroxidation assay (C) and MitoSOX in Sertoli cells and Leydig cells following GLA treatment for 24 h combined with si-NCOA4 (D). The expression levels of mitophagy-related protein (ATG5, LC3B, and SQSTM1) (E) and ferritinophagy-related protein (NCOA4 and FTH1) in Sertoli cells and Leydig cells following GLA treatment for 24 h combined with si-NCOA4 (F). Representative fluorescence confocal images of LC3B and FTH1 colocalization in Sertoli cells and Leydig cells following GLA treatment for 24 h combined with si-NCOA4 (G). Ferroptosis-related protein (ACSL4, SLC7A11, and GPX4) in Sertoli cells and Leydig cells following GLA treatment for 24 h combined with si-NCOA4 (H). “*” indicates significant difference compared with the control group (***P < 0.001). “^#^” indicates significant difference between the GLA treatment group and combined treatment group (^#^*P* < 0.05 and ^###^*P* < 0.001).

To investigate the role of ferritinophagy in ferroptosis, we designed NCOA4 knockdown experiments using siRNA-mediated silencing, and the knockdown efficiency of NCOA4 achieved 65%, further demonstrating that NCOA4 mediated GLA-induced autophagy-dependent ferroptosis (Fig. S6A). It was determined that si-NCOA4 suppressed GLA-induced increases of ferrous iron and MDA levels (Fig. 5A and B). Additionally, si-NCOA4 mitigated GLA-induced lipid peroxidation and mitochondrial dysfunction (Fig. 5C and D), as well as the decrease in MMP caused by GLA (Fig. S6B). Notably, si-NCOA4 down-regulated the LC3 ratio and ATG5 protein expression, but up-regulated SOSTM1 protein expression (Fig. 5E), thus inhibiting GLA-induced autophagy. Importantly, si-NCOA4 treatment prominently reduced NCOA4 expression, increased FTH1 expression (Fig. 5F), and promoted the co-localization of FTH1 and LC3 (Fig. 5G), inhibiting GLA-induced ferritinophagy. Furthermore, si-NCOA4 restored the GLA-induced decrease in GPX4 and SLC7A11 levels, and alleviated the up-regulation of ACSL4 (Fig. 5H), eventually inhibiting GLA-induced ferroptosis. Aforementioned data suggest that NCOA4-mediated ferritin degradation is critical for the ferroptosis of Sertoli and Leydig cells triggered by GLA.

### GLA exposure promotes ferritinophagy via activation of the AMPK-ULK1 signaling pathway in Sertoli and Leydig cells

**Fig. 6.**
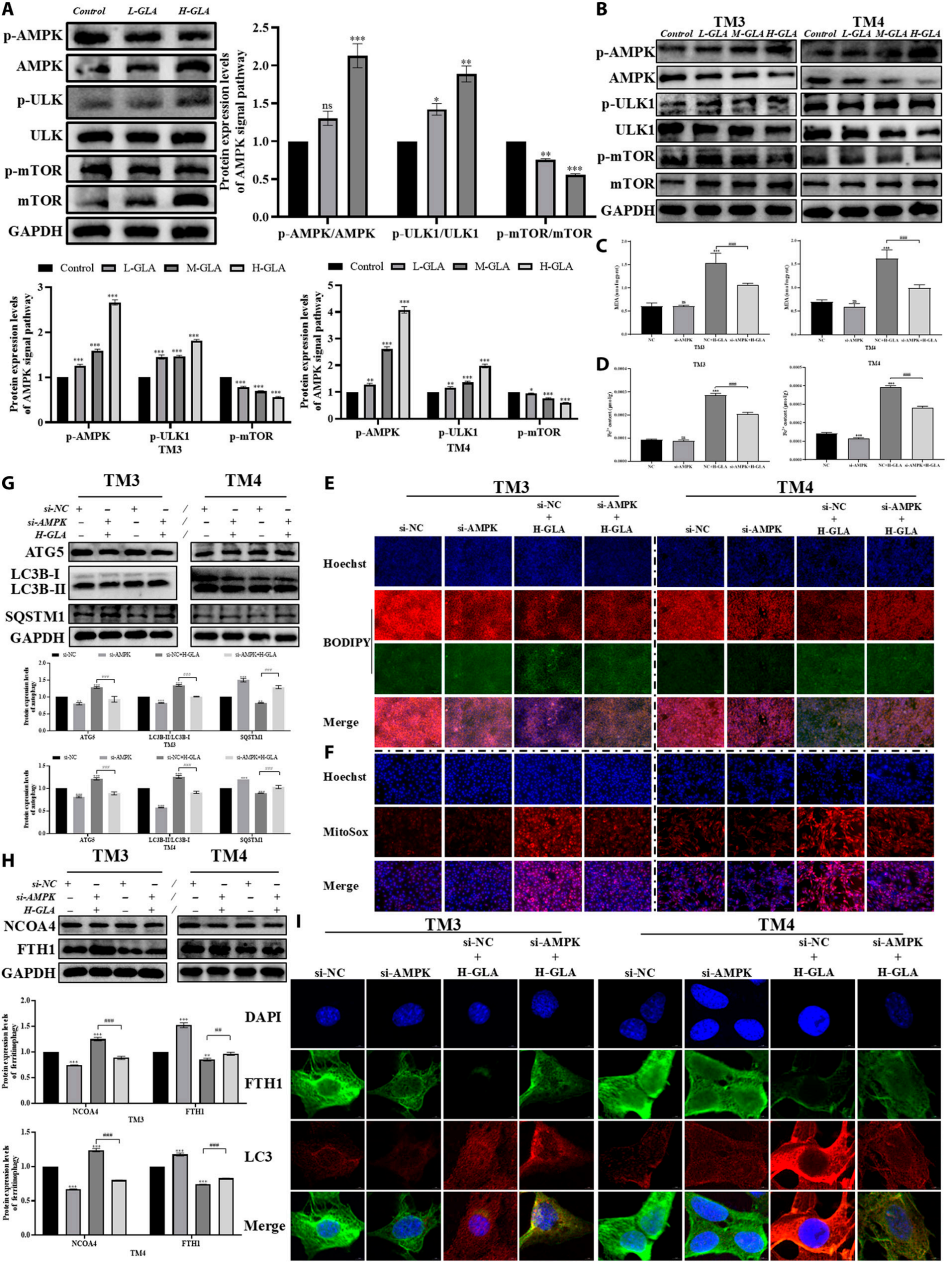
GLA exposure promotes ferritinophagy via activation of the AMPK-ULK1 signaling pathway in Sertoli cells and Leydig cells. The protein expression levels of p-AMPK, p-ULK, and p-mTOR in testes tissues (A) and Sertoli cells and Leydig cells (B). The content of Fe^2+^ (C) and MDA in Sertoli cells and Leydig cells following GLA treatment for 24 h combined with si-AMPK (D). Representative fluorescence confocal images of lipid peroxidation assay (E) and MitoSOX (F) in Sertoli cells and Leydig cells following GLA treatment for 24 h combined with si-AMPK. The expression levels of mitophagy-related protein (ATG5, LC3B, and SQSTM1) (G), ferritinophagy-related protein (NCOA4 and FTH1) (H), and LC3B and FTH1 colocalization (I) in Sertoli cells and Leydig cells following GLA treatment for 24 h combined with si-AMPK. “*” indicates significant difference compared with the control group (*P < 0.05, **P < 0.01, and ***P < 0.001). “^#^” indicates significant difference between the GLA treatment group and combined treatment group (^##^*P* < 0.01, and ^###^*P* < 0.001).

Mammalian target of rapamycin (mTOR) is a pivotal regulatory protein involved in cellular growth, proliferation, metabolism, and autophagy. mTORC1 is the main inhibitor of autophagy, inactivating the ULK1 complex by phosphorylating ATG13 and ULK1, thereby suppressing the initiation of autophagy [36]. AMP-activated protein kinase (AMPK) is a critical kinase in intracellular energy detection and autophagy signaling [37]. Unc-51-like kinase 1 (ULK1) is a pivotal kinase of autophagy initiation. The ULK1 complex activation is the first step in the autophagy process, supporting the formation of autophagosomes through the phosphorylation of autophagy-related proteins [38]. Increasing evidence suggests that AMPK and mTOR regulate the activity of the ULK1 complex through different mechanisms [39]. We observed that, compared with the control group, p-mTOR expression markedly decreased, while p-AMPK and p-ULK1 expression were dramatically elevated in the GLA-treated group (Fig. 6A and B), indicating that autophagy may be initiated through activating the AMPK-ULK1 signaling pathway in vitro and vivo.

To further confirm whether GLA-induced ferroptosis is dependent on the AMPK pathway, we conducted experiments using siRNA-mediated knockdown of AMPK with 70% knockdown efficiency (Fig. S6A). si-AMPK inhibits MDA accumulation (Fig. 6C), the increase of ferrous iron levels (Fig. 6D), lipid peroxidation (Fig. 6E), MMP loss (Fig. S6C), and mtROS generation (Fig. 6F), thereby relieving the toxic effects of GLA. Since mtROS is a key physiological activator of AMPK, in order to eliminate the influence of ROS, we pretreated the cells with the ROS scavenger butylated hydroxyanisole (BHA) before exposing them to GLA. Then, we measured the mtROS levels in the cells treated with GLA and the expression levels of protein AMPK. The results show that BHA alleviated mtROS and the inhibition of the AMPK phosphorylation process caused by GLA (Fig. S7A and B). Additionally, following si-AMPK treatment, the GLA-induced increase in autophagy (Fig. 6G) and ferritinophagy levels reverted to baseline, and the colocalization of FTH1 and LC3 is diminished (Fig. 6H and I). These observations suggest a correlation between GLA-induced ferroptosis, which is autophagy-dependent, and the activation of the AMPK-ULK1 signaling pathway.

## Discussion

Evidence suggests that environmental factors considerably influence male fertility, particularly sperm quality [40]. Since the 1990s, the widespread use of broad-spectrum herbicides like GLA has led to increased ecotoxicological risks and human exposure [41]. Emerging studies have drawn attention to the potential hazards of GLA on reproductive and developmental processes. It is well documented that GLA adversely affects sperm motility, causes malformations, and triggers immunotoxicity in zebrafish embryos [42]. Nevertheless, the effective treatments for GLA remain elusive, and it is worthy of exploring more comprehensive molecular mechanisms underlying GLA-induced toxic effects. Functional impairment of the Sertoli and Leydig cells is pivotal for the pathogenesis of testis injury. Therefore, elucidating the mechanisms by which GLA exposure affects Sertoli and Leydig cell function can provide valuable insights into GLA-induced testicular damage and reproductive toxicity. In this study, our findings showed that GLA triggered ferroptosis in testis tissue, and Sertoli and Leydig cells along with the accumulation of Fe^2+^ and lipid peroxidation, and this cell death process is governed by NCOA4-dependent ferritinophagy. Additional validation revealed that ROS and mitochondrial injury stimulated AMPK that may serve as an upstream modulator of GLA-mediated ferritinophagy. Remarkably, inhibitors of ferritinophagy, such as 3-MA, CQ, and Baf-A1, as well as ferroptosis inhibitors like DFP and Fer-1, notably restored redox balance in the GLA-exposed Sertoli and Leydig cells. Collectively, these data highlighted a previously unrecognized role of ferroptosis in the development of testicular injury caused by GLA.

Herbicide-induced tissue damage involves a range of pathophysiological responses, including mitochondrial injury, ROS production, and the release of pro-inflammatory cytokines, all of which contribute to tissue impairment and cell death [43–45]. However, the specific mechanisms underlying these effects vary by herbicide, particularly regarding their association with ferroptosis. For instance, chlorpyrifos, an organophosphate insecticide, impairs sperm parameters and reduces Sertoli/Leydig cell counts in rodents, but its reproductive toxicity primarily involves endocrine disruption and oxidative stress [16]. Although chlorpyrifos induces ROS accumulation, studies have not identified ferritinophagy or iron-dependent lipid peroxidation as key mediators. Similarly, glyphosate, another widely used herbicide, has been associated with mitochondrial dysfunction in human sperm and oxidative stress in reproductive tissues [42,43]. Some studies indicate that glyphosate’s toxicity may stem from disruptions in amino acid metabolism (e.g., glycine depletion) and gut–liver axis perturbations [46]. Notably, some toxic substances do induce ferroptosis via alternative mechanisms. For example, DEHP triggers ferroptosis in testicular tissues by targeting the transferrin receptor, disrupting iron uptake rather than ferritin degradation [27]. Similarly, diquat induces ferroptosis in spermatogonia via HO-1 up-regulation, a pathway unrelated to autophagy [28]. In contrast, several studies have reported that GLA can inhibit glutathione synthetase (GS), a key enzyme involved in ammonia detoxification and amino acid metabolism in both plants and animals, suggesting its potential toxic effects [46,47]. GS is crucial for GSH production, catalyzing the combination of γ-glutamylcysteine with glycine to form GSH. Deficiency in GS leads to reduced GSH synthesis, decreased intracellular GSH levels, heightened sensitivity to oxidative stress, and ultimately cellular damage. The System Xc^−^/GSH/GPX4 pathway represents the most thoroughly investigated mechanism to counteract ferroptosis [48]. Liedschulte et al. [49] demonstrated that ferrostatin-1 alleviated myocardial ischemia–reperfusion injury via enhancing the Xc^−^/GSH/GPX4 antioxidant axis, substantiating the clinical relevance of modulating ferroptosis. In our research, GLA treatment increased Fe^2+^ levels and decreased GPX4 and SLC7A11 protein levels; meanwhile, Fer-1 and DFP mitigated cell death by partially restoring Fe^2+^ content and GPX4/SLC7A11 expression. These data support the idea that ferroptosis is a major contributor to GLA-induced Sertoli and Leydig cell death.

Recent studies highlighted an involvement of autophagy in the cellular process of ferroptosis [34,50,51]. Our findings demonstrated that GLA triggered autophagy activation, characterized by the formation of autophagosomes and decreased SQSTM1 protein level, and increased the protein levels of ATG5 and LC3. Additionally, the autophagy in Sertoli and Leydig cells induced by GLA exposure was alleviated by treatment respectively with 3-MA, BafA1, and CQ. We subsequently investigated the relationship and causal relationship between autophagy and GLA-induced ferroptosis in Sertoli and Leydig cells. Our findings further confirmed that GLA induced autophagy in these cell types and that ferroptosis manifested as a mode of autophagic cell death. This inference is corroborated by the observation that inhibition of autophagy with autophagy inhibitors (3-MA, BafA1, and CQ) or by ATG5 knockout reduced the Fe^2+^ concentration, ferritin degradation, and lipid peroxidation upon GLA exposure. Remarkably, our results demonstrated that ATG5 knockout and treatment with 3-MA pronouncedly inhibited ferritin degradation by suppressing NCOA4 expression. In contrast, CQ and Baf-A1 promoted ferritin degradation and enhanced NCOA4 expression. Ferritinophagy is known to participate in multiple biological and pathological events, and subsistent data have been documented concerning the expression of NCOA4 during ferritinophagy. For example, earlier researches indicated that ferritinophagy is necessary for dynamic O-GlcNAcylation and the inhibition of lung injury through ferroptosis [52–55]. Besides, studies on hemochromatosis, a disorder characterized by systemic iron overload, have shown that excessive ferritinophagy in testicular tissues disrupts spermatogenesis by promoting iron-dependent oxidative stress and ferroptosis in germ cells [24,55]. NCOA4 serves as a coactivator for multiple nuclear receptors and is primarily localized within the nucleus. It interacts with FTH1 to facilitate the transfer of ferritin to autophagosomes in the process of ferritinophagy [56]. Additionally, inhibition of ferroptosis with Fer-1 and DFP decreased the expression of ATG5, LC3, and SQSTM1. These findings suggest that GLA induces ferritinophagy by promoting the formation of autophagosomes, thereby facilitating ferroptosis. Furthermore, ferritinophagy is essential for GLA-induced ferroptosis in Sertoli and Leydig cells, as demonstrated by the similar effects of NCOA4 knockdown, 3-MA treatment, as well as ATG5 knockout. Our study broadens the understanding of ferritinophagy’s functions, highlighting its role in GLA-induced dysfunction in Sertoli and Leydig cells, which contributes to testicular toxicity.

It has been established that autophagy modulators, including BECN1, CTSB, mTOR, AMPK, and DUSP1, modulate the ferroptotic response in a manner that is contingent on the specific context [35,57]. Consequently, we undertook further investigations to explore the mechanisms governing ferritinophagy. From our investigations, it was evidenced that the activation of AMPK subsequently led to the phosphorylation of ULK1, exerting a pivotal factor on the autophagy cascade. Our findings suggested that the engagement of the AMPK-ULK1 signaling axis orchestrated by GLA derived ferritinophagy and ferroptosis in Sertoli and Leydig cells. Specifically, AMPK knockdown resulted in an increase in FTH1 expression, decreased Fe^2+^ concentration, and lipid peroxidation upon GLA treatment. Additionally, we demonstrated that the downstream factor of AMPK signaling, ATG5, was up-regulated following GLA exposure. Simultaneously, ATG5 knockdown markedly enhanced the FTH1 expression and suppressed GLA-induced ferroptosis, indicating that GLA-induced ferritinophagy is mediated by the AMPK-ULK1 axis and dependent on the ATG5 process.

Recent studies have shown that mtROS serve as physiological activators of AMPK that is essential for detecting and alleviating mtROS to augment stress resilience and preserve cellular metabolic homeostasis [57]. Shafique et al. [58] reported that the AMPK/ULK1 activation by accumulation of polystyrene nanoplastics-induced lipophagy in the body was critical and led to lipid accumulation in hepatocytes. Other research has reported that increased mtROS production can enhance AMPK activation. Conversely, the application of the mitochondria-targeted antioxidant MitoQ markedly reduces the AMPK function [59]. In the current study, we noted the increase in MitoSOX levels after GLA treatment, which is consistent with the mitochondrial features associated with ferroptosis [60]. We propose that AMPK is rapidly activated in response to oxidants derived from GLA, which leads to an elevation in the Fe^2+^ levels and consequently triggers the Fenton reaction. This reaction impairs the phospholipid structure, encompassing that of mitochondrial membranes. Prior studies have suggested that deprivation of cysteine augments the tricarboxylic acid cycle and enhances electron transport chain activity, resulting in the hyperpolarization of the MMP and the accumulation of lipid peroxides, ultimately inducing ferroptosis [61]. Our investigation revealed that the amino acid exchange mechanism of System Xc^−^ was compromised by GLA, as evidenced by reduced SLC7A11 expression, hindering cysteine uptake into the cells. Eventually, this led to a depletion of GSH, following by an increase in MMP. The mitochondria were continuously exposed to ROS generated by the Fenton reaction, causing MMP collapse and further endogenous ROS production, which activates the AMPK signaling and exacerbates ferroptotic death in Sertoli and Leydig cells. These findings suggested that mtROS-induced ferritinophagy served as a precursor phenomenon of GLA-mediated ferroptosis, which is consistent with current research indicating that mitochondria exert a crucial and active function in cysteine deficiency-triggered ferroptosis [62].

## Materials and Methods

### Study design

The conduct of all animal experiments strictly adhered to established protocols and gained formal ratification from the Institutional Animal Care and Use Committee (IACUC) at Jiangxi Agricultural University. Specific pathogen-free male Kunming mice, aged 5 weeks, were obtained from Changsha Tianqin Biotechnology Co., Ltd. (Changsha, China). The mice were housed under standard laboratory conditions with controlled environmental parameters (a temperature of 22 ± 2 °C, a relative humidity of 50% ± 5%, and a 12-h light/12-h dark cycle) and provided ad libitum access to food and water throughout the 1-week acclimatization period and the entire experimental duration. Following acclimatization, a total of 72 mice were evenly distributed into 3 distinct groups, each comprising 24 animals. The control group was administered distilled water, while the treatment groups received GLA (CAS No. 77182-82-2, purity ≥ 97.93%) via intragastric administration at concentrations of 20 and 60 mg/kg body weight per day, respectively. Daily measurements of food consumption and body weight were meticulously recorded. After 35 days of treatment, mice were humanely euthanized. Immediately afterward, testes were collected to minimize post-mortem changes, with the euthanasia-to-sampling interval strictly controlled within 5 min. Dissection was performed under sterile conditions: the abdominal cavity was opened along the midline, testes were carefully excised with sterile forceps, and the surrounding adipose and connective tissues were removed to prevent contamination. For subsequent analyses, testes were processed per experimental needs: histological samples were fixed in 4% paraformaldehyde (PFA) for 24 h; biochemical samples were snap-frozen in liquid nitrogen immediately after dissection and stored at −80 °C until analysis; and TEM samples were fixed in 2.5% glutaraldehyde at 4 °C. The entire experimental protocol was vetted and sanctioned by the IACUC at Jiangxi Agricultural University (JXAULL-2023-11-10). Throughout the treatment period, utmost care was taken to minimize any potential discomfort for the animals, ensuring their welfare was prioritized.

TM3 and TM4 cells, procured from Northeast Agricultural University (Harbin, China), were maintained in a 1:1 mixture of Ham’s F12 and Dulbecco’s Modified Eagle Medium (F12/DMEM), supplemented with 10% fetal bovine serum, 100 U/ml penicillin, and 100 μg/ml streptomycin, in a humidified environment with 5% CO₂ at 37 °C. The TM3 and TM4 cells were pretreated with GLA for 24 h. Subsequently, autophagy was inhibited by separately adding 3-MA, CQ, and BafA1 or knocking down ATG5 and AMPK; ferroptosis was induced by adding Erastin; and ferroptosis was inhibited by separately adding ferrostatin-1 and DFP or knocking down NCOA4, as required by the experimental design. In addition, cells were pretreated with BHA, an ROS scavenger, for 3 h, followed by treatment with GLA for 24 h [63]. The drug concentration is shown in Table 1.

**Table 1.** Drug concentration

Dosage	Leydig cell (TM3)	Sertoli cell (TM4)
L-GLA (μM)	253.4	166.2
M-GLA (μM)	337.9	207.8
H-GLA (μM)	506.9	277
3-MA (μM)	90	60
CQ (μM)	4	0.5
Baf-A1 (nM)	4	12
DFP (μM)	4	6
Fer (nM)	200	200
Erastin (nM)	100	200
BHA (μM)	50	10
si-ATG5 (nM)	150	150
si-NCOA4 (nM)	200	200
si-AMPK (nM)	200	200

### Reagents and antibodies

GLA (SG9020) and Ferrous Ion Content Assay Kit (BC5415) were provided by Beijing Solarbio Science & Technology Co., Ltd. (Beijing, China). Acridine Orange Stain kit (PS0063) was provided by Beijing JinMing Biotechnology Co., Ltd. (Beijing, China). Sperm nucleoprotein staining solution (aniline blue method; R30404) was supplied by Shanghai Yuanye Bio-Technology Co., Ltd. (Shanghai, China). Ferrostatin-1 (Fer-1; S7243), DFP (S4067), 3-methyladenine (3-MA; NSC66389), chloroquine diphosphate (CQ; S4157), bafilomycin A1 (Baf-A1; S1413), Erastin (S7242), and BHA (E1925) were provided by Selleck (Shanghai, China). Cell Counting Kit-8 (CCK-8; GK10001) was provided by Glpbio (California, USA). YO-PRO-1and PI (C1075S) and Hoechst 33342 (C1028) were furnished by Beyotime Institute of Biotechnology (Shanghai, China). MitoSOX Red (HY-D1055) was furnished by MedChemExpress (Shanghai, China). JC-1 Mitochondrial Fluorescent Probes (J4001) was provided by UElandy (Suzhou, China). RNAFit-RNA siRNA Transfection Reagent (HB-RF-1000), NC siRNA, siRNA against ATG, siRNA against NCOA4, and siRNA against AMPK were all purchased from HanBio Technology (Shanghai, China).

GPX4 antibody (T56959F), SLC7A11 antibody (T57046), ACSL4 antibody (T510198F), NCOA4 antibody (TD4255F), FTH1 antibody (T55648F), ULK1 antibody (T56902), and ATG5 antibody (T55766) were obtained from Abmart Shanghai Co., Ltd. (Shanghai, China). AMPK antibody (WL02254), p-AMPK antibody (WL05103), p-mTOR antibody (WL03694), mTOR antibody (WL02477), LC3 antibody (WL01506), and SQSTM1 antibody (WL02385) were provided by Wanleibio (Shenyang, China). P-ULK1 antibody (S556) was provided by Abways (Shanghai, China). GAPDH antibody (60004-1-lg) was provided by Proteintech Group, Inc. (Wuhan, China).

### Evaluation of sperm quality

According to previous reports [64], the caudal epididymides were minced in 0.9% saline solution at 37 °C to discharge and spread semen. The sperm was stained by Acridine Orange Stain kit and Sperm nucleoprotein staining solution following the instructions. Images were captured under a fluorescence microscope (Nikon, Japan).

### PAS staining of testicular tissue

The specific operation steps were according to a previously reported experimental methodology [65]. Fresh testicular tissue was immersed promptly in a 4% PFA solution to undergo fixation, followed by dehydration, embedding, and sectioning. The samples underwent PAS staining, were rinsed with tap water, dehydrated, became transparent, sealed, and then scanned using a digital section scanner. The scoring of spermatogenesis was conducted in accordance with the Johnsen’s method executed in 10 various fields within every section.

### Detection of cell viability

To evaluate cell viability, the CCK-8 method was employed. Cells were plated in 96-well microplates at a density of 2 × 10^3^ cells per well, with 100 μl of culture medium in each well. After the necessary treatments and growth period, 10 μl of CCK-8 reagent was added to each well and incubated at 37 °C for 40 min, adhering to the protocol provided by the manufacturer. The optical density of each well was subsequently determined at 450 nm using a microplate reader. To guarantee the consistency and statistical significance of the results, the experiment was repeated 3 times, with each condition tested in technical triplicates.

### YO-PRO-1 and PI double staining

Cells were plated onto a 6-well culture dish and exposed to GLA. The medium was then aspirated, and the cells were rinsed with DMEM. Next, 1 ml of the YO-PRO-1/PI staining solution was introduced to each well. Following incubation at 37 °C for 20 min, fluorescence staining was visualized under a fluorescence microscope. Green fluorescence (Ex/Em = 491/509 nm) indicated YO-PRO-1 positivity, whereas red fluorescence (Ex/Em = 535/617 nm) corresponded to PI positivity.

### Detection of MMP, mtROS, and lipid peroxidation

MMP was detected using the JC-1 mitochondrial fluorescent probe, while mtROS levels were assessed with the MitoSOX Red probe. The BODIPY 581/591 C11 probe was utilized to evaluate lipid peroxidation. Following treatment, cells were subjected to incubation with JC-1 (10 μg/ml), Mito Tracker (1 μM), and BODIPY 581/591 C11 (1 μM) for 30 min at 37 °C. Nuclei were stained with Hoechst 33342 for 5 min at 37 °C. Fluorescence signals were assessed via fluorescence microscopy and quantified using ImageJ software.

### siRNA and transfection

When the cells reached a confluence of 30% to 50%, transfection was performed using 150 nM siRNA with the RNAFit transfection reagent for 12 h, adhering to the guidelines provided by the manufacturer. Following the initial steps, the cells were transferred to a fresh complete DMEM/F12 medium and allowed to grow for an additional 12-h period. GLA treatment was subsequently administered to the cells for the subsequent experiments. For these experiments, 3 specific siRNA sequences targeting ATG5, NCOA4, and AMPK were designed and synthesized. The exact sequences are detailed in Table 2**.**

**Table 2.** The sequences of the siRNA in this study

si-RNA name	Sense (5′–3′)	Antisense (5′–3′)
*ATG5*-si-2	CCAUCAACCGGAAACUCAUTT	AUGAGUUUCCGGUUGAUGGTT
*NCOA4*-si-1	CGAUCUCAUCUAUCAGCUUAATT	UUAAGCUGAUAGAUGAGAUCGTT
*Prkaa1*-si-1	CACGAGUUGACCGGACAUAAATT	UUUAUGUCCGGUCAACUCGUGTT

### Transmission electron microscopy

TEM assays were conducted according to the protocol detailed in the previous section [66,67]. Testis samples and cells were first fixed with 2.5% glutaraldehyde, washed with 0.1 M phosphate buffer, and then post-fixed with 1% osmium tetroxide. After another rinse, the samples were dehydrated using acetone and embedded in an appropriate embedding medium. Ultrathin sections were stained with 3% uranyl acetate and lead citrate for ultrastructural examination. The resulting micrographs were captured using a JEM-1400 Flash transmission electron microscope.

### Immunofluorescence staining

Immunofluorescence staining was carried out on both testicular tissue and cultured cells according to established procedures [27]. In particular, the samples were subjected to fixation with 4% PFA and permeabilization using 0.5% Triton X-100. Post-blocking with 5% goat serum, the specimens were sequentially treated with primary antibodies, followed by secondary antibodies, and then stained with 4',6-diamidino-2-phenylindole (DAPI). Thereafter, imaging was performed utilizing a Zeiss LSM 510 META laser scanning confocal microscope (Carl Zeiss, Oberkochen, Germany).

### Detection of iron in cells and testicular tissue

The labile Fe^2+^ concentration in cells and testicular tissue was measured using a Ferrous Iron Content Detection Kit, following the guidelines provided by the manufacturer. Testicular tissue and cells were mixed with the appropriate volume of Reagent 1 in proportion, followed either by homogenization in an ice bath or by ultrasonication. Subsequently, the mixture was centrifuged at 10,000 *g* for 10 min at 4 °C, and the resulting supernatant was kept on ice prior to further analysis. Subsequently, the standard solution was prepared adhering to the outlined procedures, and the samples were mixed with the required reagents prior to being loaded into a 96-well plate. The absorbance was measured at 593 nm utilizing an enzyme-linked instrument. Subsequently, the standard curve was generated, and the outcomes were computed based on this curve.

### Determination of antioxidant capacity

Testicular tissues were homogenized in PBS on ice, and cells were lysed in PBS using sonication. Following centrifugation, collection of the supernatant was performed. The level of MDA and GSH was assessed with corresponding kits conforming to the manufacturer’s suggestions. A microplate reader was employed for these measurements.

### Western blotting

To analyze proteins from testicular tissues and cell samples, the extracted proteins were solubilized in a cold buffer containing radio-immunoprecipitation assay buffer (RIPA), phenylmethylsulfonyl fluoride (PMSF), and protease inhibitors. The protein concentration was assessed using the BCA method. The protein samples were then subjected to sodium dodecyl sulfate-polyacrylamide gel electrophoresis (SDS-PAGE) for separation and transferred to polyvinylidene fluoride (PVDF) membranes. After blocking, the membranes were incubated overnight at 4 °C with primary antibodies. The membranes were further incubated with secondary antibodies for 40 min at room temperature. The protein bands were detected with an ECL detection kit and their intensities were quantified using ImageJ software. For additional methodological details, see previous studies [68].

### Statistical analysis

Statistical analysis was performed using GraphPad Prism version 10.0 and SPSS software version 26.0 (IBM Corporation, Armonk, NY, USA). To compare data across different groups, independent-samples *t* tests and one-way analysis of variance were employed. All data are presented as mean values with standard deviation (SD). A *P* value of less than 0.05 was considered indicative of a statistically significant difference.

## Data Availability

The data are freely available upon request.
