# Supplementary material for: AMPK-ULK1-Mediated Ferritinophagy Drives Ferroptosis in GLA-Induced Testicular Toxicity
Source: Research (Wash D C). 2025 Jun 25;8:0860. doi: 10.34133/research.0860 (PMC12377500; doi:10.34133/research.0860)
Supplement: Supplementary 1 — Figs. S1 to S7 [file research.0860.f1.docx]

**
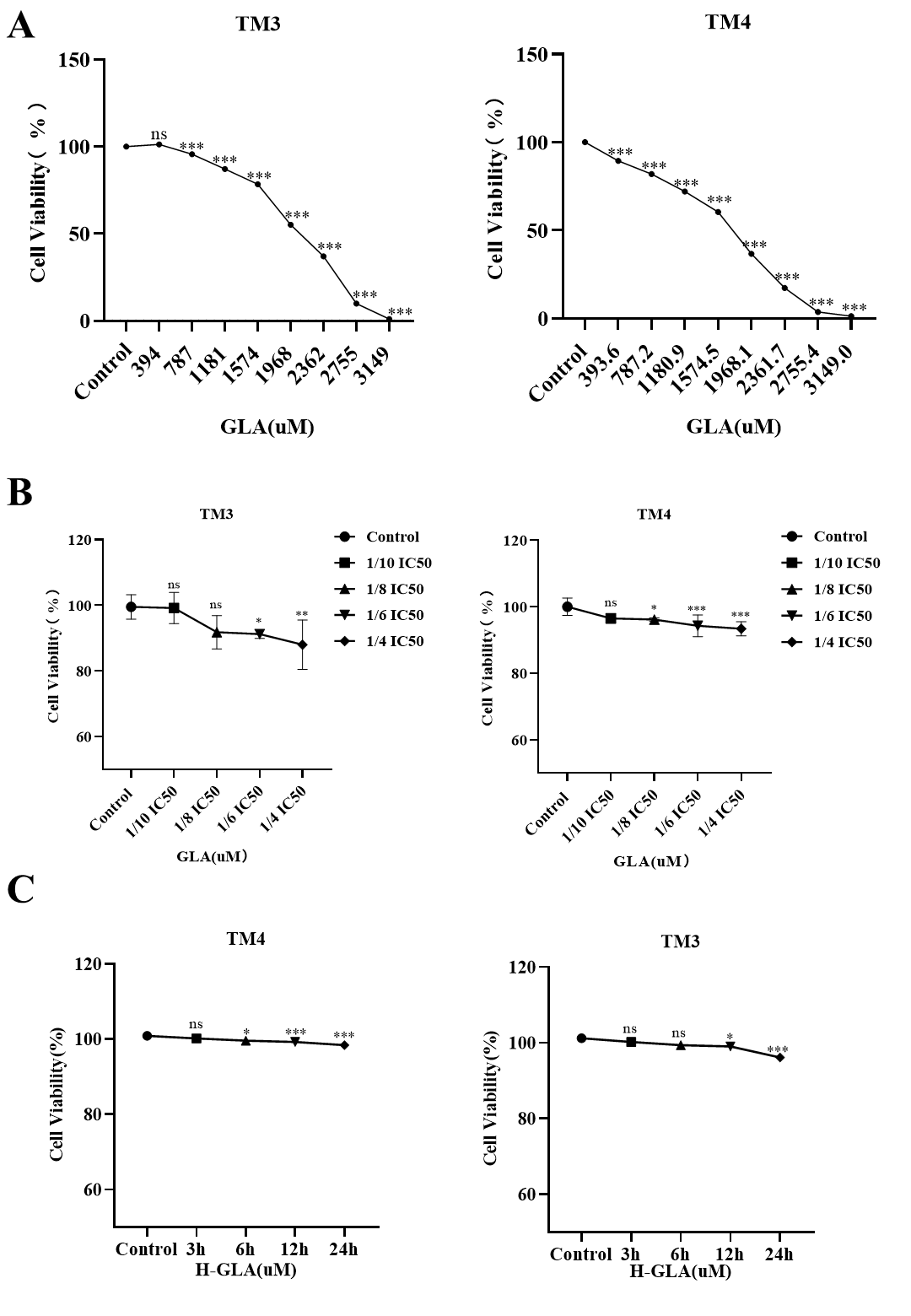
**

**Figure. S1.** Exposure to different GLA concentrations for a 24-hour period to measure the cell viability for Sertoli cells and Leydig cells to measure the IC50 concentration (n = 6) (A). Sertoli cells and Leydig cells were treated with GLA concentrations of 1/10, 1/8, 1/6, and 1/4 of the IC50 for 24 h to assess cell viability (n = 6) (B). Sertoli cells and Leydig cells were treated with GLA highest concentration to cells for 0, 6, 12, and 24 h to assess cell viability (n = 6) (C).


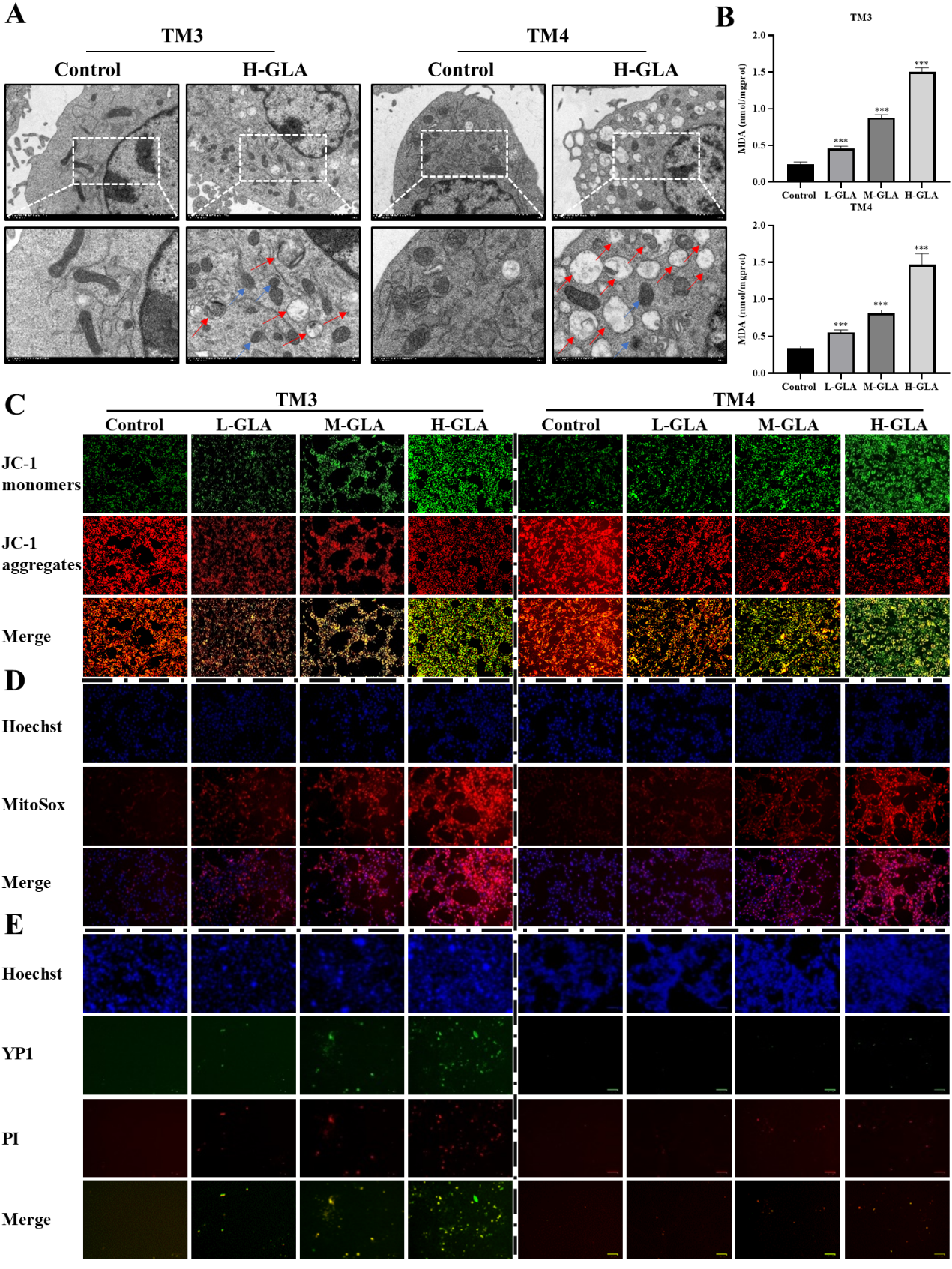


**Figure. S2.** GLA exposure induces Sertoli cells and Leydig cells damage. Representative TEM images of mitochondria in Sertoli cells and Leydig cells treated with 277 μM or 506.9 μM GLA for 24 h, blue arrows indicate mitochondria with severely disrupted cristae, red arrows indicate vacuolated mitochondria with autophagic vacuole (A). MDA content (B). Representative fluorescence confocal images of JC-1 (C). Representative fluorescence confocal images of mROS with MitoSox dye (D). Representative fluorescence confocal images of cytoactive (E).

**
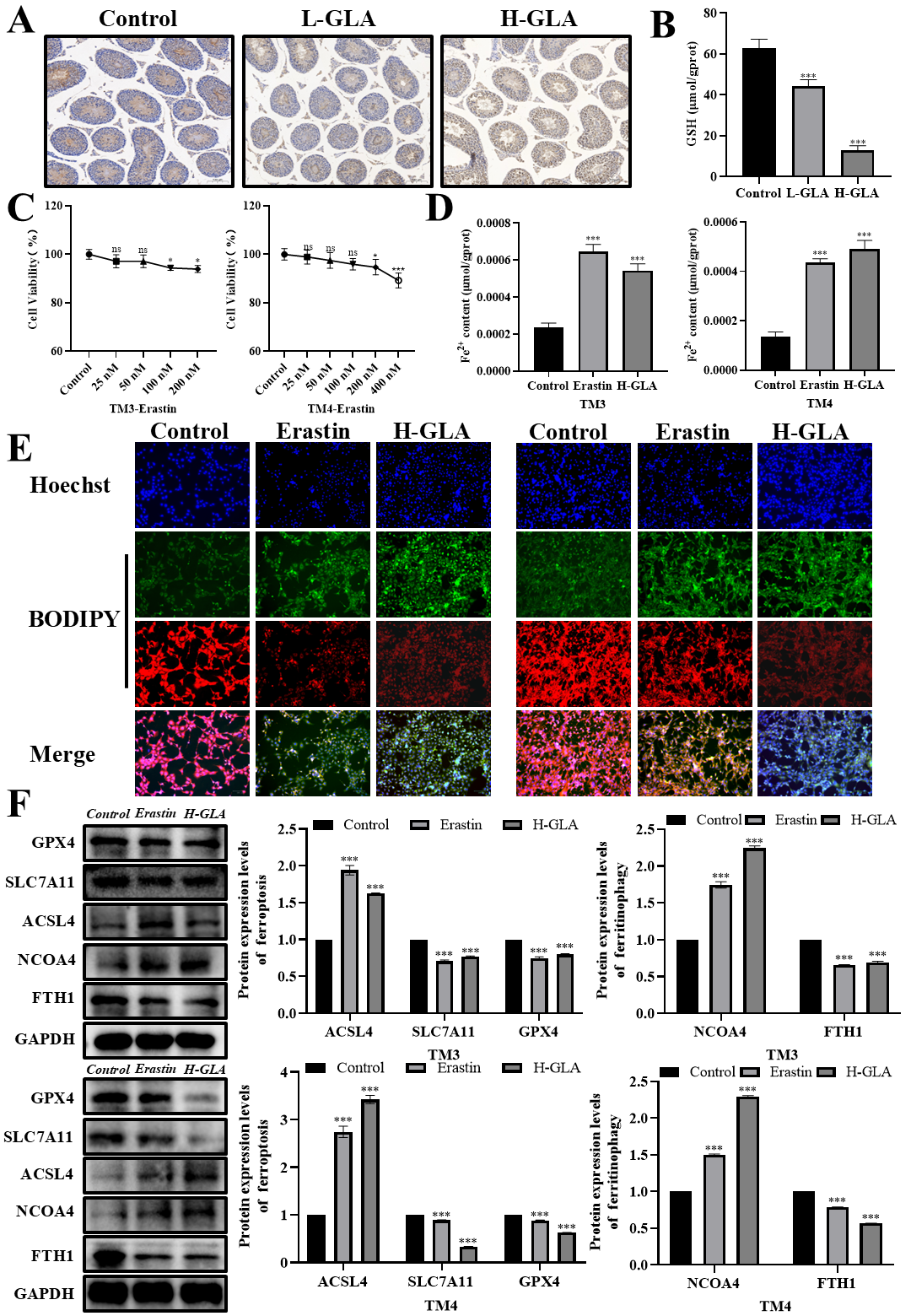
**

**Figure. S3.** GLA exposure induces ferroptosis. The immunohistochemical staining of GPX4 in testicular tissue (A). GSH level in testicular tissue (B). Sertoli cells and Leydig cells were treated with different concentrations of Erastin for 24 h to assess cell viability (n = 6) (C). The Fe^2+^ content of Sertoli cells and Leydig cells (D). The protein expression levels of GPX4, SLC7All, ACSL4, NCOA4 and FTH1 in Sertoli cells and Leydig cells following H-GLA treatment for 24 h combined with Erastin (100 nM or 200 nM) (E). Representative fluorescence images of lipid peroxidation assay in Sertoli cells and Leydig cells following H-GLA treatment for 24 h combined with Erastin (100 nM or 200 nM) (F)


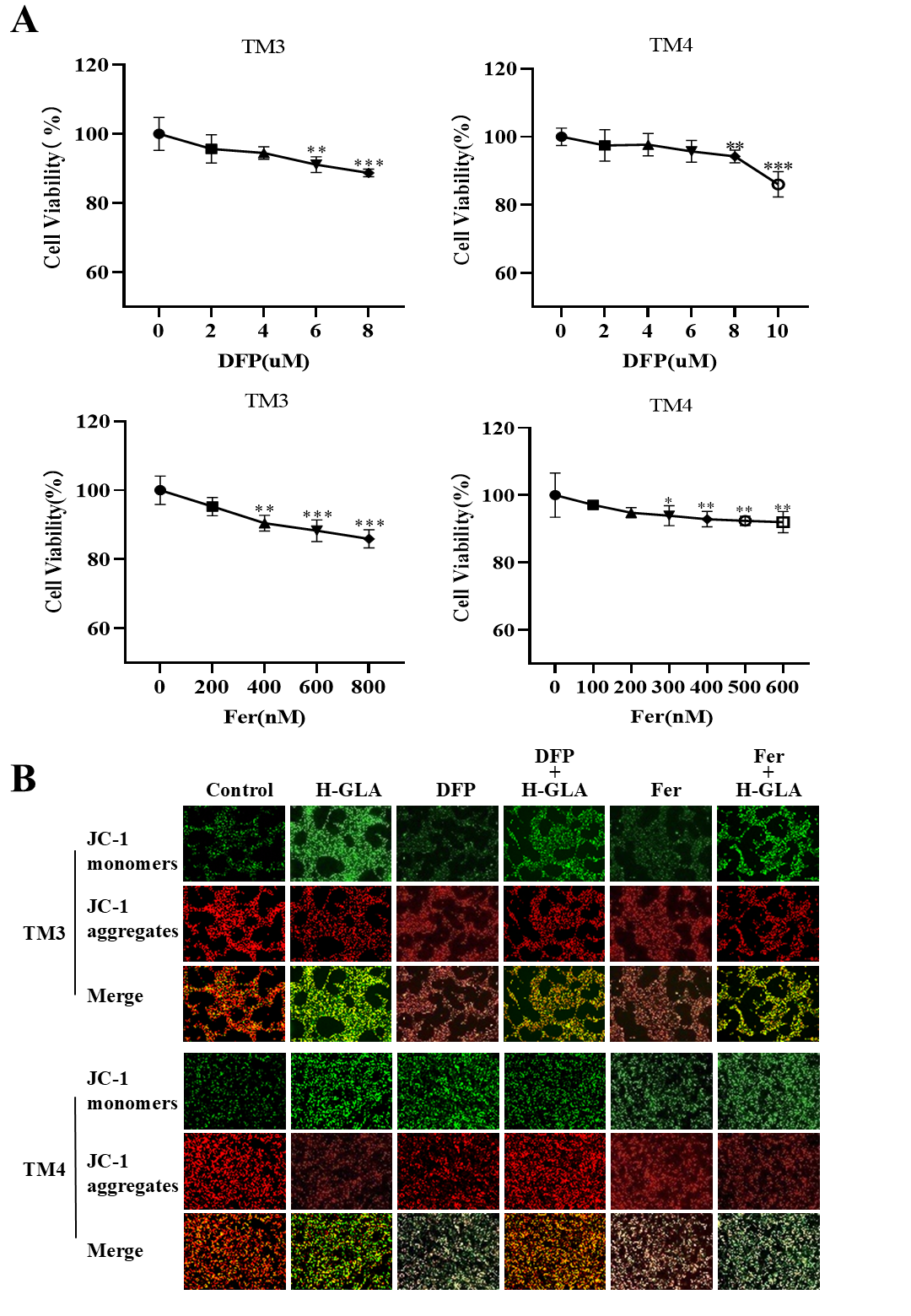


**Figure. S4.** Sertoli cells and Leydig cells were treated with different concentrations of Fer or DFP for 24 h to assess cell viability (n = 6) (A). Representative fluorescence confocal images of JC-1 in Sertoli cells and Leydig cells following GLA treatment for 24 h combined with Fer or DFP (B).


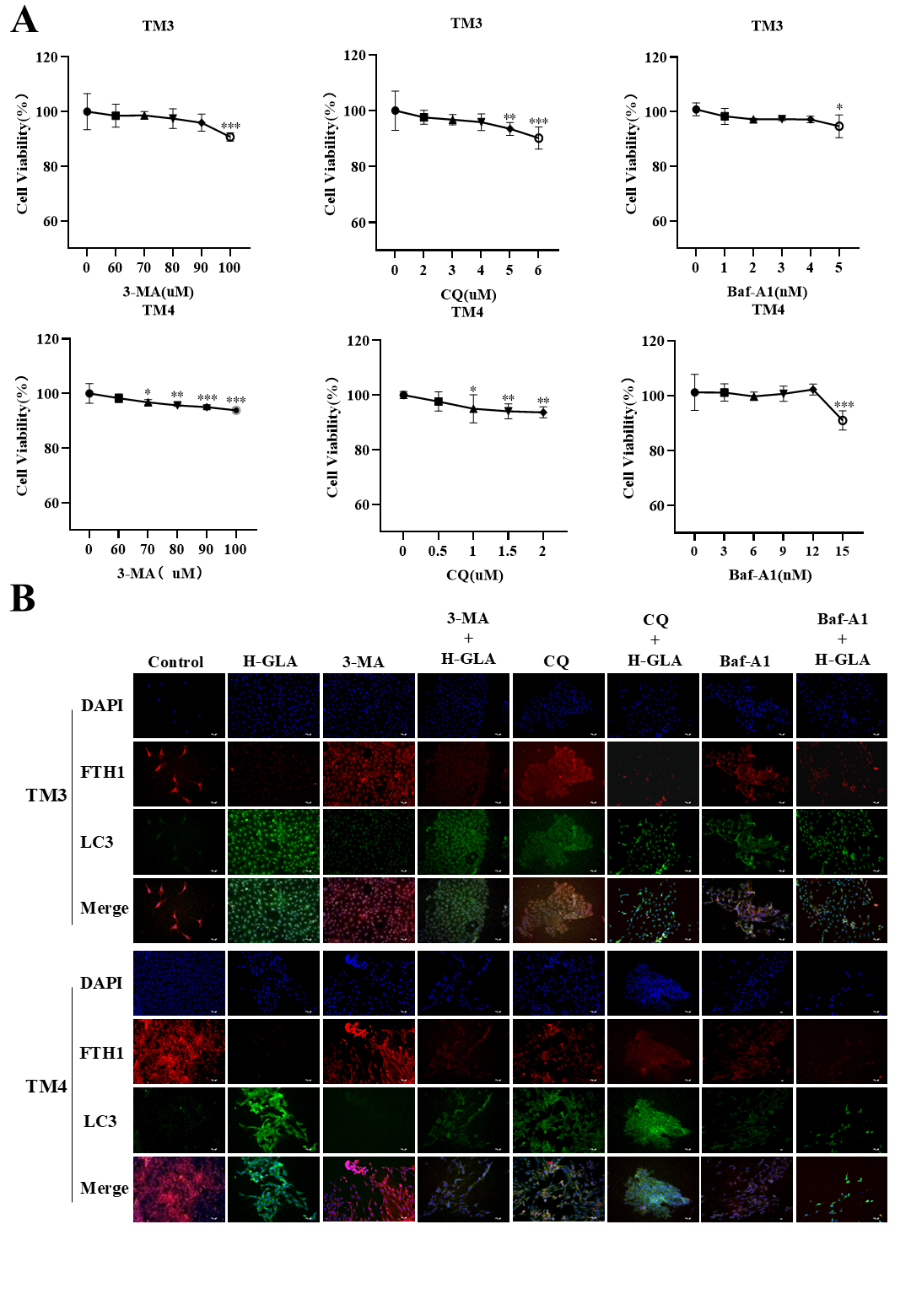


**Figure. S5.** Sertoli cells and Leydig cells were treated with different concentrations of 3-MA, CQ, or Baf-A1 for 24 h to assess cell viability (n = 6) (A). Representative fluorescence confocal images of LC3B and FTH1 colocalization in Sertoli cells and Leydig cells following GLA treatment for 24 h combined with3-MA, CQ, or Baf-A1 (B).


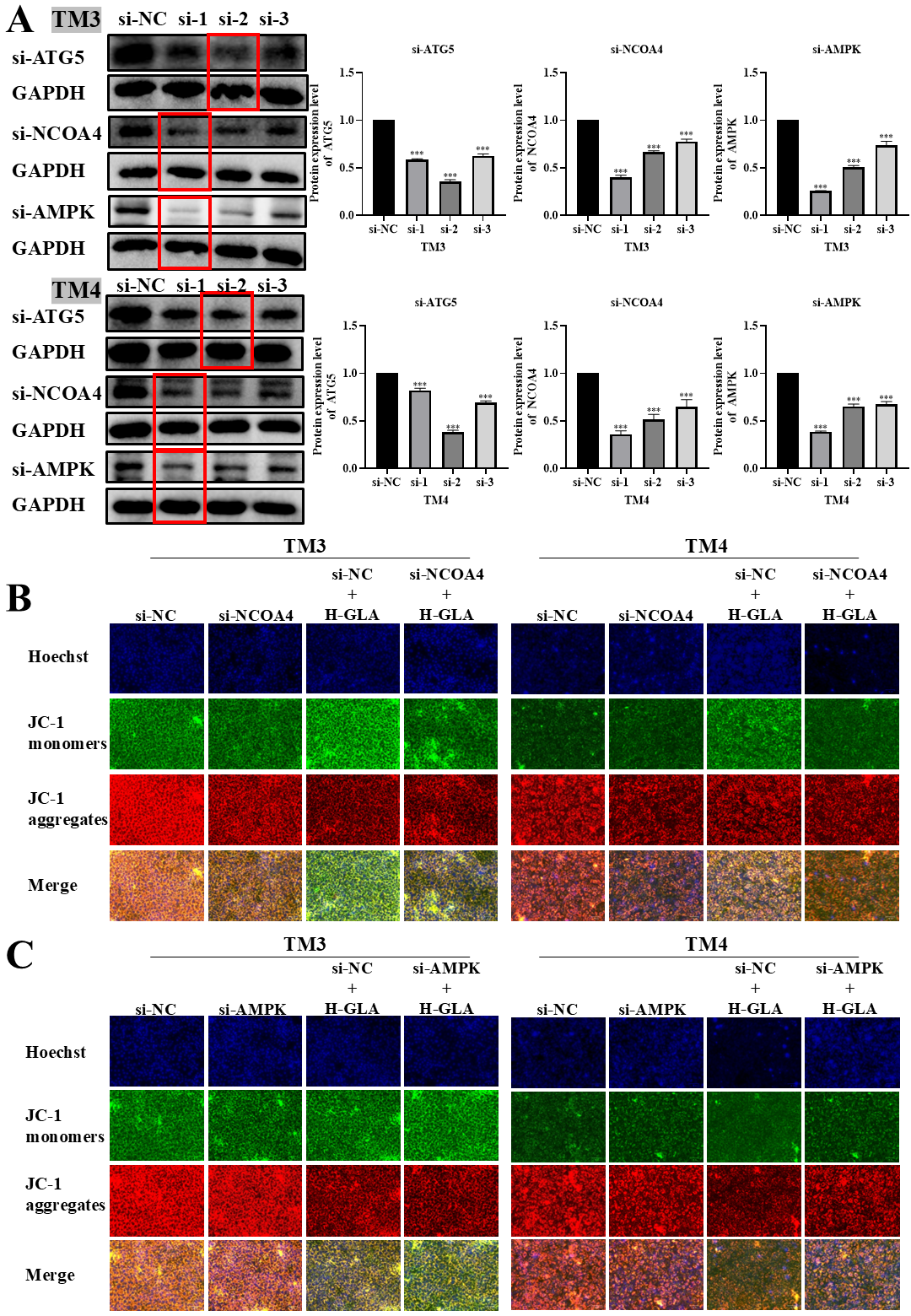


**Figure. S6.** The knockdown efficiency of ATG5, NCOA4, and AMPK in Sertoli cells and Leydig cells (A). Representative fluorescence confocal images of JC-1 in Sertoli cells and Leydig cells following GLA treatment for 24 h combined with si-NCOA4 (B). Representative fluorescence confocal images of JC-1 in Sertoli cells and Leydig cells following GLA treatment for 24 h combined with si-AMPK (C).


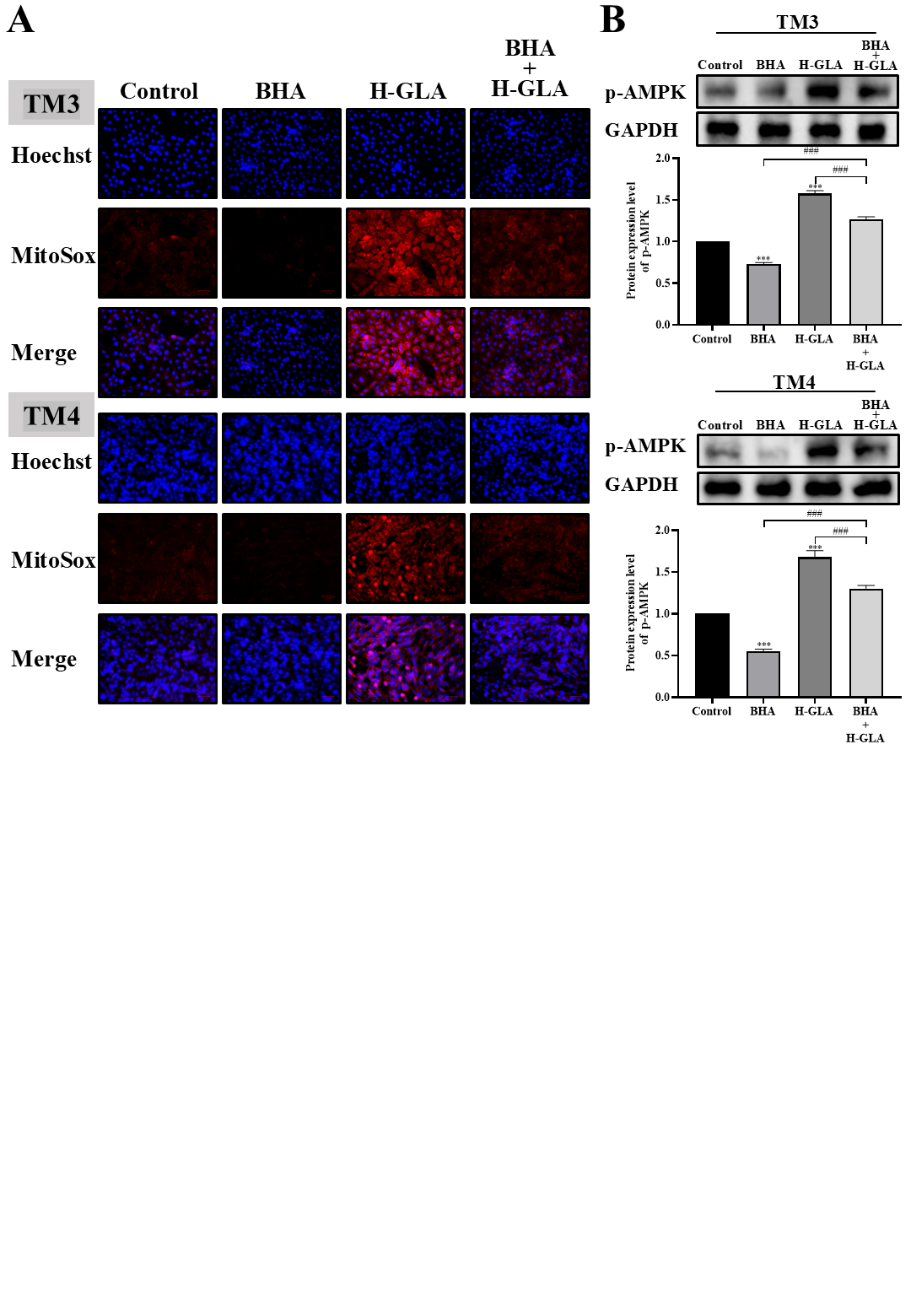


**Figure. S7.** Representative fluorescence confocal images of MitoSox in Sertoli cells and Leydig cells following BHA pretreatment for 3 h and GLA treatment for 24 h (A). The protein expression levels of p-AMPK in Sertoli cells and Leydig cells following BHA pretreatment for 3 h and GLA treatment for 24 h (B)
